# Cadmium Impairs Human GnRH Neuron Development: Mechanistic Insights into Reproductive Dysfunction

**DOI:** 10.3390/ijms27031221

**Published:** 2026-01-26

**Authors:** Giulia Guarnieri, Jacopo J. V. Branca, Rachele Garella, Letizia Lazzerini, Flavia Mencarelli, Francesco Palmieri, Paolo Comeglio, Matteo Becatti, Mario Maggi, Massimo Gulisano, Alessandra Pacini, Roberta Squecco, Annamaria Morelli

**Affiliations:** 1 Department of Experimental and Clinical Medicine, University of Florence, 50134 Florence, Italy; giulia.guarnieri@unifi.it (G.G.); jacopojuniovalerio.branca@unifi.it (J.J.V.B.); rachele.garella@unifi.it (R.G.); lazzerini.letizia08@gmail.com (L.L.); flavia.mencarelli@unifi.it (F.M.); francesco.palmieri@unifi.it (F.P.); massimo.gulisano@unifi.it (M.G.); alessandra.pacini@unifi.it (A.P.); roberta.squecco@unifi.it (R.S.); 2 Department of Experimental and Clinical Medicine, Imaging Platform, University of Florence, 50134 Florence, Italy; 3 Department of Experimental and Clinical Biomedical Sciences “Mario Serio”, University of Florence, 50134 Florence, Italy; paolo.comeglio@unifi.it (P.C.); matteo.becatti@unifi.it (M.B.); mario.maggi@unifi.it (M.M.)

**Keywords:** GnRH neurons, heavy metal, cell migration, reproduction, gap junctions, fertility, pollution

## Abstract

There is increasing evidence that exposure to environmental toxicants may impact fertility, especially during critical windows of reproductive axis development. Hypothalamic gonadotropin-releasing hormone (GnRH) neurons, essential for puberty onset and fertility, originate from the olfactory placode and migrate toward the hypothalamus during development, making them particularly vulnerable to environmental insults. Cadmium (Cd), a widespread heavy metal, is well known for its gonadotoxicity, but its impact on human hypothalamic neuron development remains unclear. Using human fetal GnRH neuroblasts (FNCB4) we investigated the effects of Cd exposure on their morpho-functional and developmental features. Cd induced oxidative stress and COX2 mRNA upregulation, indicative of inflammatory pathway activation, which was accompanied by reduced cell migration and downregulation of motility-related genes. These effects were associated with F-actin disassembly and altered expression of adhesion molecules. Electrophysiological analyses showed that Cd altered membrane potential, increased capacitance and permeability, and disrupted gap junctional communication, as also confirmed by connexin-43 delocalization. Moreover, Cd significantly reduced the expression of specific GnRH neuronal markers, suggesting impaired functional maturation. Overall, our findings provide the first evidence that Cd may interfere with mechanisms crucially involved in human GnRH neuron development, adding new mechanistic insights into the comprehension of how early-life exposure to Cd may contribute to fertility concerns.

## 1. Introduction

Although safety regulations on pollutant exposure have been implemented, environmental contaminants remain a constant part of daily life and pose serious risks to human health, contributing significantly to various diseases. Beyond carcinogenic effects that have been well established for the most hazardous categories of environmental pollutants [1], there is increasing evidence that some of them have played a role in the worsening of reproductive health, as registered in recent decades [2]. A number of studies have reported that certain pollutants may negatively impact the reproductive system by several mechanisms, including oxidative stress, direct genetic damage, and proapoptotic actions exerted at the gonadal/germ cell level. In addition, hormone-mediated effects of several environmental contaminants have been recognized, since they may act as endocrine disrupting chemicals (EDCs). EDCs represent a continuously growing group of compounds able to interfere with the endocrine system and thereby with many physiological functions, including reproduction [3].

Within the wide list of environmental pollutants, the heavy metal cadmium (Cd) has been classified by the World Health Organization (WHO) as one of the ten chemicals of major public health concern due to its persistence in the environment, prolonged biological half-life, and toxicological effects [4]. Cd is predominantly released into the environment through anthropogenic activities, including fossil combustion, tobacco smoking, mining, and the manufacture of batteries, pigments, or fertilizers. In the general population, chronic exposure to Cd occurs primarily through ingesting contaminated food, particularly vegetables, crops, and shellfish, with secondary contributions from inhalation of polluted air and contaminated water [5,6]. Once absorbed, Cd persists in the human body for several decades, with a biological half-life estimated between 20 and 30 years, leading to a progressive accumulation in various organs and long-term manifestation of toxic effects, even at low concentrations [7].

Over recent decades, several studies have linked Cd exposure to reproductive health concerns. A growing body of evidence in both human and experimental studies supports the detrimental effects of Cd on both human male and female reproduction [8]. Moreover, Cd belongs to the heavy metal category of EDCs with no known physiological role that could mimic or inhibit the actions of endogenous hormones, especially during critical windows of reproductive system development [9]. A positive correlation with primary ovarian insufficiency and a negative correlation with semen quality have been demonstrated for Cd in population case studies [10,11]. In female mice, exposure to increasing concentrations of Cd was negatively associated with a reduction in fertilization rate [12]. In addition, endometrial concentrations of heavy metals, including Cd, have been identified in the etiology of unexplained infertility [13].

A major challenge in investigations into the effects of environmental contaminants on reproductive health arises from the complexity of the reproductive system itself, which involves finely balanced cooperation between the brain and other endocrine organs, including gonads, constituting the hypothalamic–pituitary–gonadal (HPG) axis. The hypothalamus secretes the gonadotropin-releasing hormone (GnRH) within the hypophysial portal system to reach the anterior pituitary and stimulate the release of gonadotropins, follicle-stimulating hormone (FSH), and luteinizing hormone (LH). In turn, FSH and LH regulate gonadal maturation, gamete production, and sex steroid hormone secretion in both sexes [14]. With a feedback mechanism, gonadal hormones act at the hypothalamic and pituitary levels in order to directly or indirectly regulate GnRH activity. Hence, GnRH neurons, scattered within the preoptic area of the hypothalamus, are the key regulators of the HPG axis, and their activity is required for initiating gonadal maturation at puberty and then for maintaining reproductive function over adulthood.

Although several data suggest a primary effect of Cd on male [9] and female gonads [15], it is increasingly recognized that Cd may also target the central components of the HPG axis at the brain level. In fact, a recent study in patients with idiopathic hypogonadotropic hypogonadism reported higher Cd blood levels than in matched healthy controls [16]. Although in that study a relationship between Cd levels and measured androgens was not found, in another study in aging males, a negative correlation between Cd levels with both free T and DHEAS was observed [17]. In animal models, Cd exposure has been shown to impair the secretion of gonadotropins, alter hypothalamic gene expression, and induce histopathological changes in key hypothalamic regions involved in reproductive control [18,19]. Most interestingly, a recent study demonstrated that Cd exposure decreased the expression of hypothalamic GnRH mRNA, leading to disruption of the HPG axis in a prepubertal rat model [19]. Additionally, Cd exposure can affect neurodevelopment, altering differentiation and maturation of cells in the brain, especially progenitor cells, as well as causing significant cell subpopulation shifts in almost all types of brain cells [20].

This is particularly relevant for GnRH neurons, which are exceptionally vulnerable due to their unique developmental path. Indeed, GnRH neurons originate from progenitor cells outside the brain in the olfactory placode, and during fetal development, differentiate and migrate along the route of the olfactory nerves to reach their final destination within the hypothalamus, where at the time of puberty onset they start to drive the HPG axis [21]. Successful migration and morphofunctional maturation of GnRH neurons are critical steps for the correct achievement of reproductive function. Any alteration in these processes during fetal life may have long-term consequences [22], resulting in infertility and clinical conditions of varying severity, such as congenital hypogonadotropic hypogonadism (CHH). The most severe form of CHH is Kallmann syndrome, which is caused by incomplete fetal migration of GnRH-secreting neurons and is characterized by anosmia, gonadotropin deficiency, and pubertal failure [23]. In this regard, exposure to Cd during fetal development is biologically relevant, since it tends to accumulate in placental tissue and alter its barrier function, as demonstrated by detectable Cd levels in umbilical cord blood, confirming transplacental transfer [24,25]. The risk of increased placental permeability to Cd appears to be further elevated with increasing maternal age, body mass index (BMI), or body weight [26]. Once transferred, fetal exposure to Cd may increase the risk of developmental disorders, including alterations of the reproductive system [27].

Despite the growing evidence of Cd toxicity at the central level, studies directly examining the effects of Cd in the different steps of GnRH neuron development, including proliferation, differentiation, and migration of the olfactory precursors, have not been clearly identified, especially in humans. Most current knowledge derives from rodent studies, thus restricting the understanding of human-specific mechanisms and limiting the accuracy of reproductive health risk evaluations.

To address this gap, the present in vitro study was aimed at investigating whether Cd exposure may directly interfere with the differentiation and function of human GnRH neuroblasts, taking advantage of the long-term FNCB4 cell culture originally derived from the olfactory epithelium of an 8-week-old human fetus and well characterized as a model of human GnRH neuroblasts [28,29]. The effects of Cd in FNCB4 cells were investigated in terms of cytotoxicity, phenotype changes, and functional response.

## 2. Results

### 2.1. Cd-Induced Cytotoxicity in FNCB4 Cells

**Figure 1:**
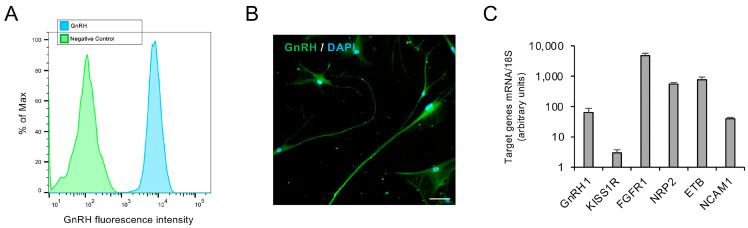
Phenotypic characterization of FNCB4 neuroblasts. (**A**) Representative overlaid histogram of FACS analysis for GnRH protein expression in FNCB4 cells (light-blue peak). The negative control is the green peak. (**B**) Representative immunofluorescence image of GnRH-positive cells (green); DAPI-counterstained nuclei; scale bar: 100 µm. (**C**) Relative mRNA expression of target genes by qRT-PCR. Genes were normalized over the 18S ribosomal RNA subunit, used as the housekeeping gene, and reported as means ± SEM (*n* = 9).

The identity of FNCB4 cells as GnRH neurons was confirmed here, in accordance with data already published. In particular, the FNCB4 cells are GnRH-positive, as demonstrated by flow cytometry analysis (Figure 1A) and immunocytochemistry (Figure 1B), along with the expression of genes characterizing the GnRH neuroblast phenotype (Figure 1C). Indeed, as detected by qRT-PCR, FNCB4 expressed GnRH1 along with genes known to be implicated in GnRH neuron migration (FGFR1, NRP2, ETB, NCAM1) and functional response (KISS1R) to the main physiological regulator kisspeptin (Figure 1C).

**Figure 2:**
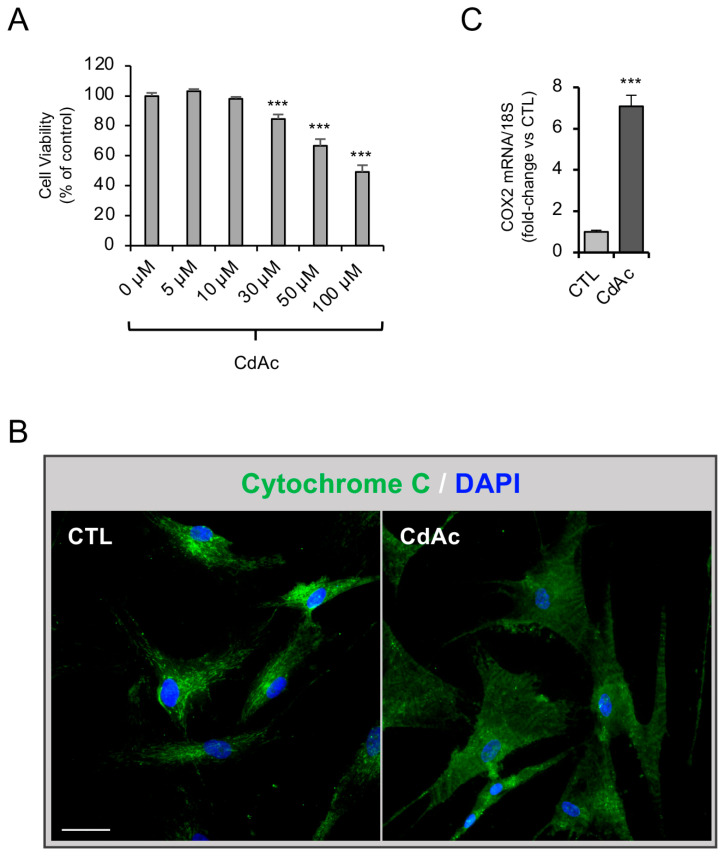
Cadmium cytotoxicity in FNCB4 cells. (**A**) MTT assay of CdAc-treated FNCB4 cells at different concentrations (0, 5, 10, 30, 50, and 100 µM) for 24 h. Cell viability is expressed as the percentage of viability over untreated (control, CTL) cells (0 µM CdAc), taken as 100%, and reported as means ± SEM (*n* = 3). *** *p* < 0.001 vs. CTL. (**B**) Representative immunofluorescence images of cytochrome C localization (green) in untreated (CTL) and CdAc-treated (CdAc, 10µM) cells for 24 h. DAPI-counterstained nuclei; scale bar: 50 µm. (**C**) mRNA expression of cyclooxygenase 2 (*COX2*) in untreated (CTL, light-gray box) and CdAc-treated (10 μM for 24 h; dark-gray box) cells. Data were normalized over 18S ribosomal RNA subunit, used as the housekeeping gene. Results are expressed as fold change vs. CTL and reported as means ± SEM (*n* = 6). *** *p* < 0.001 vs. CTL.

In order to determine the effect of Cd on cell viability, we first performed an MTT assay after exposing FNCB4 cells to increasing concentrations of Cd acetate (CdAc; 0, 5, 10, 30, 50, and 100 µM) for 24 h. Dose selection was based on cell viability screening to identify a subcytotoxic concentration for subsequent experiments in the FNCB4 cellular model. As shown in Figure 2A, a significant dose-dependent decrease in cell viability was observed for higher CdAc concentrations (30, 50, 100 µM). Based on these results, the subtoxic dose of 10 µM CdAc was selected, as it also reflects exposure conditions consistent with chronic human bioaccumulation and placental transfer alterations [30,31] and has been widely employed in human neuronal in vitro cadmium-related toxicity research [32,33,34]. Notably, IC_50_ values for Cd vary considerably across different neuronal models and cytotoxic assays, with some studies reporting toxic effects at or close to the 10 µM dose [35,36,37,38], highlighting the model-dependent nature of Cd sensitivity.

It is well known that Cd toxicity is often associated with inflammation and reactive oxygen species (ROS) production in the central nervous system [39,40]. To evaluate its effects on human GnRH neurons, we analyzed CdAc cytotoxicity by performing immunostaining against cytochrome C. As shown in Figure 2B, FNCB4 cells treated with CdAc (10 µM, 24 h) expressed a diffuse cytoplasmic signal, representing a massive cytochrome C release from mitochondria, which normally occurs as a cell stress alarm induced by ROS generation. We next investigated the CdAc-induced inflammatory response by qRT-PCR analysis of COX2 mRNA expression (Figure 2C). A significant increase in COX2 mRNA expression was detected in treated cells compared to untreated ones (*p* < 0.001).

### 2.2. Cd Affects the Migratory Ability of FNCB4 Cells

**Figure 3:**
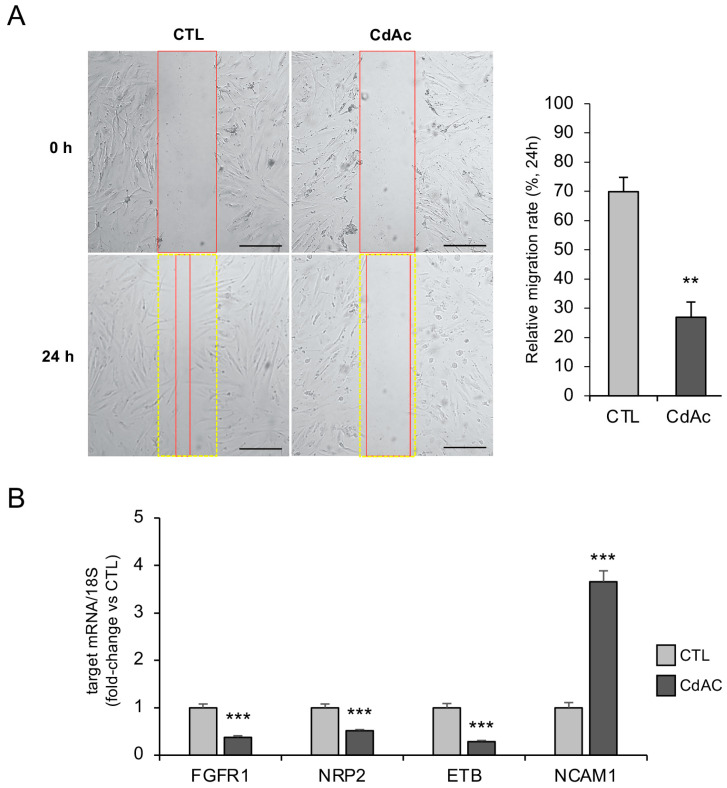
Cd effects on FNCB4 cell migration. (**A**) Representative phase-contrast microscope images and bar graph showing quantitative analysis of wound-healing migration assay in FNCB4 cells exposed (CdAc) or not (control, CTL) to CdAc (10 µM) for 24 h. The percentage of cell migration was determined by the rate of cells covering the scratch area 24 h after wounding. Yellow dotted lines mark the scratch area at 0 h, while red lines indicate the areas lacking cells at 0 and 24 h. Results are expressed as the percentage of cell migration rate and reported as means ± SEM (*n* = 3). ** *p* < 0.01 vs. CTL. Scale bar: 400 µm. (**B**) mRNA expression of migration-related target genes in untreated (CTL, light-gray box) and CdAc-treated (10 μM for 24 h; dark-gray box) cells. Data were normalized over 18S ribosomal RNA subunit, used as the housekeeping gene. Results are expressed as fold change vs. untreated cells (CTL) and reported as means ± SEM (*n* = 6). *** *p* < 0.001 vs. CTL.

Given that cell migration is a crucial process for the proper development of GnRH neurons, we first investigated the effect of Cd on the migratory ability of FNCB4 neuroblasts. As demonstrated by the wound-healing migration assay, CdAc (10 µM) exposure for 24 h significantly reduced the cell migration rate of FNCB4 compared to untreated cells (*p* < 0.01; Figure 3A). The alteration of the migratory properties in CdAc-treated FNCB4 was also confirmed by the significant reduction in the mRNA levels of genes crucially involved in GnRH neuron migration, such as FGFR1, NRP2, and ETB (all *p* < 0.001 vs. CTL; Figure 3B). Interestingly, CdAc exposure significantly increased (*p* < 0.001) the mRNA expression of NCAM1, which is involved in cell adhesion and GnRH neuron migration mechanisms (Figure 3B).

### 2.3. Effect of Cd on Cytoskeletal Remodeling in FNCB4 Cells

**Figure 4:**
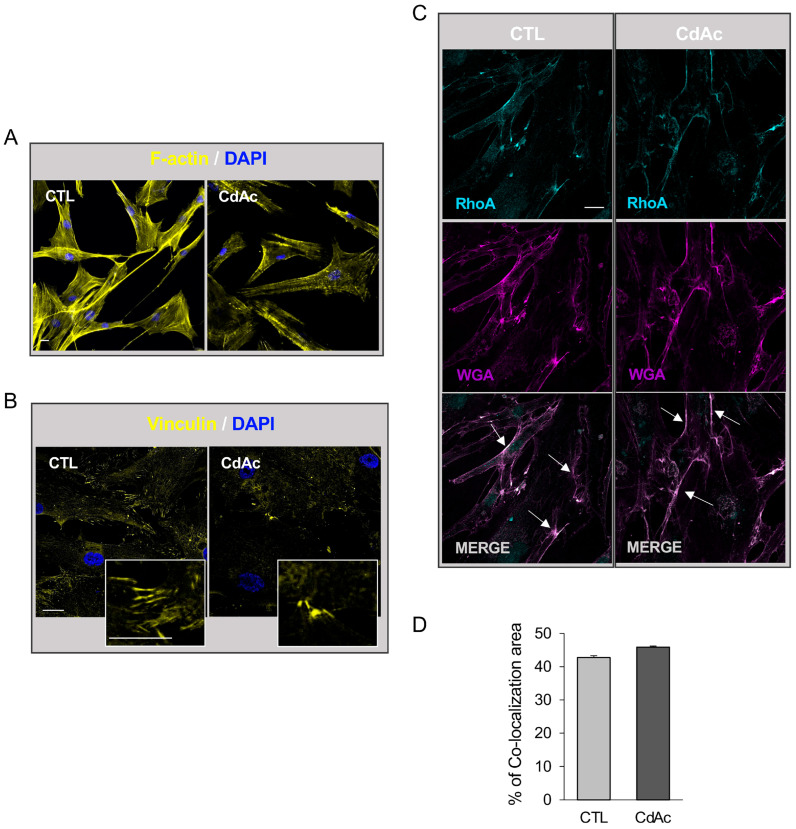
Cd effects on cytoskeletal organization in FNCB4 cells. (**A**) Representative immunofluorescence images of actin filaments (F-actin, yellow) in untreated (control, CTL) and CdAc-exposed FNCB4 cells (10 µM) for 24 h. DAPI-counterstained nuclei. Scale bar: 20 µm. (**B**) Representative immunofluorescence images of vinculin (yellow) showing focal adhesions in untreated (control, CTL) and CdAc-exposed FNCB4 cells (10 µM) for 24 h. The square inserts show the magnified region. DAPI-counterstained nuclei. Scale bars: 20 µm. (**C**) Representative fluorescence images showing immunolocalization of RhoA (cyan) at the plasma membrane co-stained with wheat germ agglutinin (WGA, magenta) in untreated (control, CTL) and CdAc-exposed FNCB4 cells (10 µM) for 24 h. The bottom row displays merged images where the white signal indicates areas of RhoA and WGA colocalization, highlighted by arrows. Scale bar: 20 µm. (**D**) Histogram representing the percentage of RhoA/WGA co-localizing area normalized by total area occupied by cells in FNCB4 cells exposed (CdAc) or not (control, CTL) to CdAc (10 µM) for 24 h. Results are reported as means ± SEM (*n* = 3). Corresponding raw grayscale TIFF images are provided in Supplementary Material S1 for reference.

Since changes in cell morphology and adhesion processes are crucial for several cellular functions, including cell migration, we next investigated whether CdAc could act on the rearrangement of the cytoskeleton in FNCB4. Fluorescence imaging of phalloidin staining for detecting filamentous actin (F-actin) revealed a uniformly dense network of actin stress fibers in untreated cells with well-organized cytoplasmatic actin bundles and cortical actin filaments beneath the plasma membrane (Figure 4A). In contrast, 24 h of exposure to CdAc led to actin filament fragmentation, affecting global cytoskeletal organization in FNCB4 cells, as demonstrated by a dot-like signal and globally lower fluorescence of phalloidin staining (Figure 4A). The dynamic of focal adhesion was also affected by CdAc. The immunofluorescence images of vinculin, a component of focal adhesions and adherin junctions, showed a reduced number of focal adhesion points and decreased fluorescence intensity of the vinculin-positive signal in CdAc-treated FNCB4 cells compared to untreated cells (Figure 4B).

To further investigate the effects of Cd on mechanisms implicated in cell migration, we analyzed the RhoA/ROCK pathway, which is crucially involved in regulating cytoskeletal remodeling. Since upon activation, RhoA translocates from the cytosol to the plasma membrane, we performed dual immunostaining using antibodies against RhoA and the membrane marker WGA. In untreated cells, RhoA was mainly localized to the plasma membrane and Cd treatment (10 μM for 24 h) did not change RhoA membrane positivity, as quantitatively analyzed in terms of the percentage of colocalization area, normalized by the entire area covered by cells (Figure 4C, *p* = 0.703). These data were confirmed by Pearson correlation coefficient (PCC) analysis, with the PCC value of untreated cells (mean PCC = 0.677 ± 0.027) being not statistically different in comparison to the Cd-exposed cells (mean PCC = 0.606 ± 0.025).

### 2.4. Cd Affects Cell-to-Cell Communication

**Figure 5:**
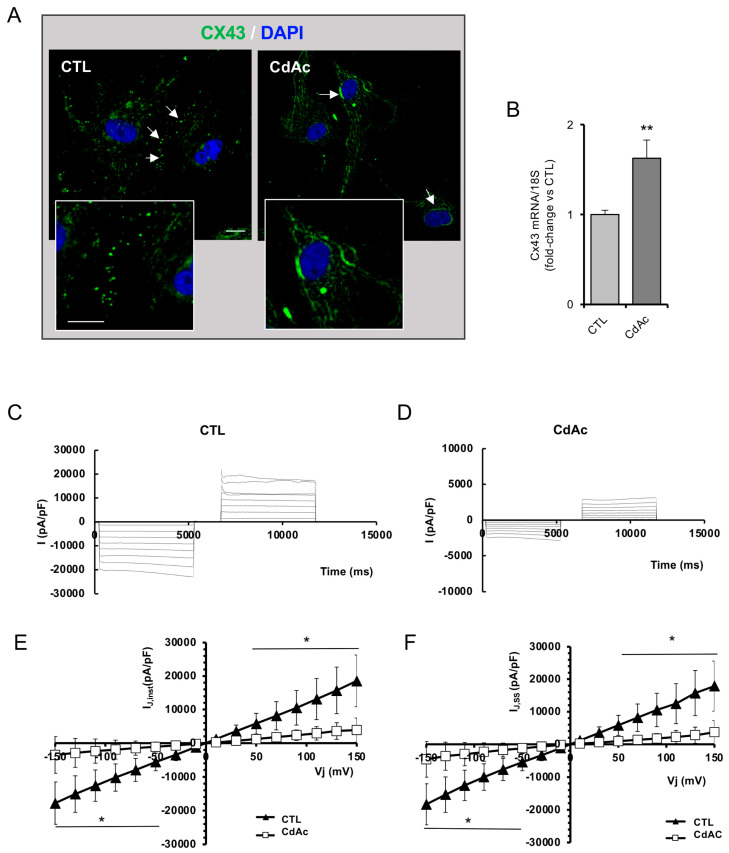
Effect of cadmium on gap junctions. (**A**) Representative immunofluorescence images of Cx43 (green) in untreated (control, CTL) and CdAc-exposed FNCB4 cells (10 µM) for 24 h. Arrows point to Cx43 distribution at the cell–cell surface in untreated (CTL) FNCB4 cells and at cytoplasmic and perinuclear Cx43 localization in CdAc-treated cells. The right panel is a magnification of the areas pointed at by the arrows. DAPI-counterstained nuclei. Scale bar: 20 µm. (**B**) mRNA expression of *CX43* in untreated (CTL, light-gray box) and CdAc-treated (10 μM for 24 h; dark-gray box) FNCB4 cells. Data were normalized over the 18S ribosomal RNA subunit, used as the housekeeping gene. Results are expressed as fold change vs. untreated cells (CTL) and reported as means ± SEM (*n* = 9). ** *p* < 0.01 vs. CTL. (**C**,**D**) Representative time courses of the current flowing from one cell to the adjacent one through GJs recorded from an untreated (CTL) (**C**) and from a Cd-treated FNCB4 cell pair (**D**). (**E**,**F**) I–V plot showing the voltage dependence of the I_J_,inst (**E**) and I_J_,ss (**F**). Filled triangles are data from untreated cells (CTL), (*n* = 3); open squares are data from CdAc treatment (*n* = 3). Data are reported as means ± SD. * *p* < 0.05 vs. CTL.

Migration of GnRH neurons requires proper cell-to-cell communication to achieve a coordinated process that is primarily regulated by gap junction function. To evaluate this function, we first performed an immunolocalization analysis of the gap junction protein connexin 43 (Cx43) in FNCB4 cells. As reported in Figure 5A, untreated cells showed a punctate and discontinuous expression of Cx43 within the plasma membrane, especially on the cell-to-cell contact side, with some Cx43 signal also present in the cytoplasm. In contrast, CdAc exposure determined a mainly cytoplasmatic and perinuclear distribution of Cx43 with no correct gap junction structure at the cell-to-cell surface. Interestingly, CdAc significantly induced the mRNA expression of Cx43, suggesting a compensatory mechanism in FNCB4 cells in response to the toxic insult (Figure 5B).

To further confirm this finding, we also assessed the effect of CdAc on gap junctions with electrophysiological studies. The transjunctional currents flowing through the gap junctions turned out to be reduced in amplitude in the cell pairs treated with CdAc in comparison with the untreated cell pairs (Figure 5C,D), both for the instantaneous current amplitude I_J,inst_ and for that measured at the steady state, I_J,ss_. The I–V plots reporting the mean current values evoked by any transjunctional voltage (Vj) for all of the experiments confirmed that the current flowing through gap junctions in CdAc-treated cells was significantly smaller than that recorded in control, suggesting a reduced gap junction functionality for any Vj applied (Figure 5E,F). Of note, this result is in line with the observed reduction in gap junction structures at the cell-to-cell surface in CdAc-exposed cells.

### 2.5. Cd Affects Electrophysiological Properties of FNCB4 Cells

**Figure 6:**
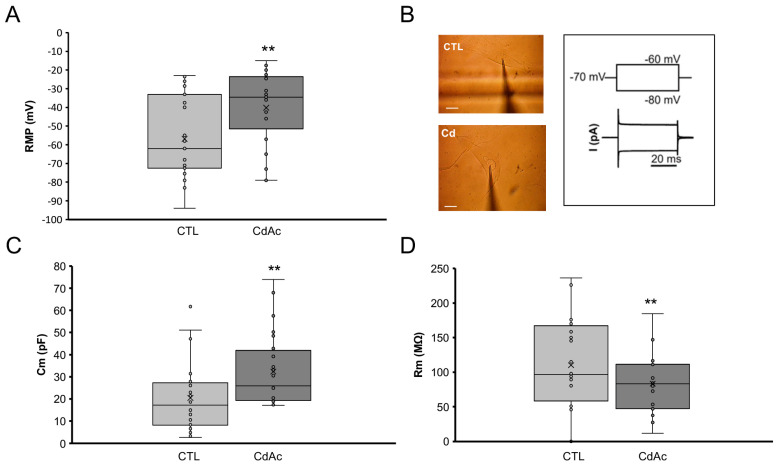
Effects of Cd on FNCB4 passive membrane properties. (**A**) RPM values (in mV) recorded in the current clamp mode from control (CTL, untreated cells, *n* = 27) and CdAc-treated FNCB4 cells (CdAc, *n* = 25). (**B**) Representative CTL cell (top) and CdAc-treated cell (bottom) impaled by the patch pipette. Scale bar 20 µm; inset: voltage-clamp protocol and illustrative passive current responses. (**C**) Cell capacitance values, Cm (in pF) (CTL *n* = 26; CdAc, *n* = 24). (**D**) Membrane resistance, Rm, values (in MΩ) (CTL *n* = 17; CdAc, *n* = 19). Data are reported as means ± SD. ** *p* < 0.01 vs. CTL.

To better characterize whether Cd treatment affected the functional properties of FNCB4 cells, we performed electrophysiological measurements. We found that CdAc (10 µM, 24 h) significantly affected the resting membrane potential (RMP) compared to control (untreated cells), causing a statistically significant depolarization (*p* < 0.01; Figure 6A), which led to altered cell excitability. Moreover, the evaluation of membrane passive currents evoked by the voltage-clamp pulse protocol (Figure 6B) indicated significant modifications.

In particular, in CdAc-treated cells, we observed a clear increase in cell capacitance, Cm (*p* < 0.05; Figure 6C). Since this parameter is related to cell membrane area, this observation suggested a surface increase compared to untreated cells. Moreover, CdAc-treated cells showed reduced membrane resistance (Rm; Figure 6D), indicative of increased permeability.

**Figure 7:**
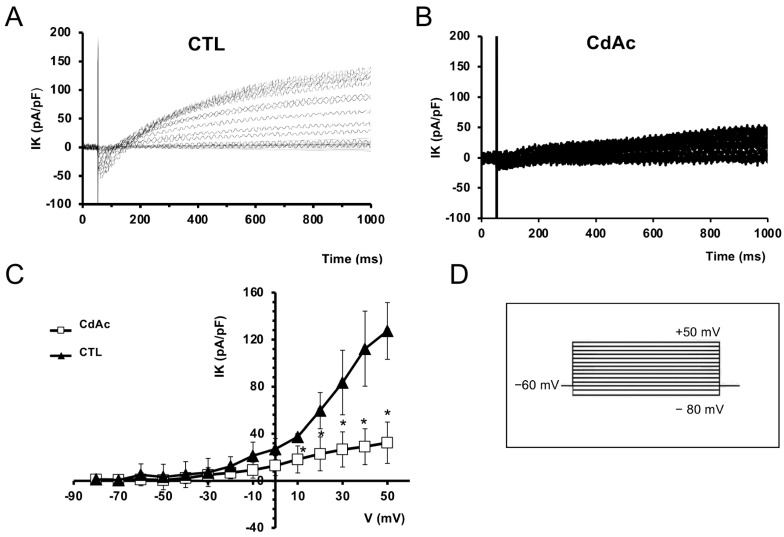
Effects of Cd on K^+^ currents in FNCB4 cells. (**A**,**B**) Representative K^+^ current traces (in pA) recorded from an untreated ((**A**), CTL) and a CdAc-treated cell (**B**). (**C**) I–V plot (in pA/pF) related to all the experiments (CTL, filled triangles, *n* = 3; CdAc, open squares, *n* = 5). For voltage pulses positive to +10 mV, current values are significantly different (* *p* < 0.05). Data are expressed as means ± SD. (**D**) Voltage step pulse protocol of stimulation.

In addition, the analysis of transmembrane ion currents showed significant differences between the cells cultured in the two conditions: control cells usually exhibited larger K^+^ currents than CdAc-treated cells (Figure 7A,B). The I–V relationship related to all the experiments done confirmed this behavior, showing significant differences between the two conditions (Figure 7C; *p* < 0.05).

### 2.6. Cadmium Affects FNCB4 Phenotype

The impact of CdAc on GnRH neuroblast phenotype and function was also assessed by analyzing the expression of the specific lineage markers of GnRH neurons.

**Figure 8:**
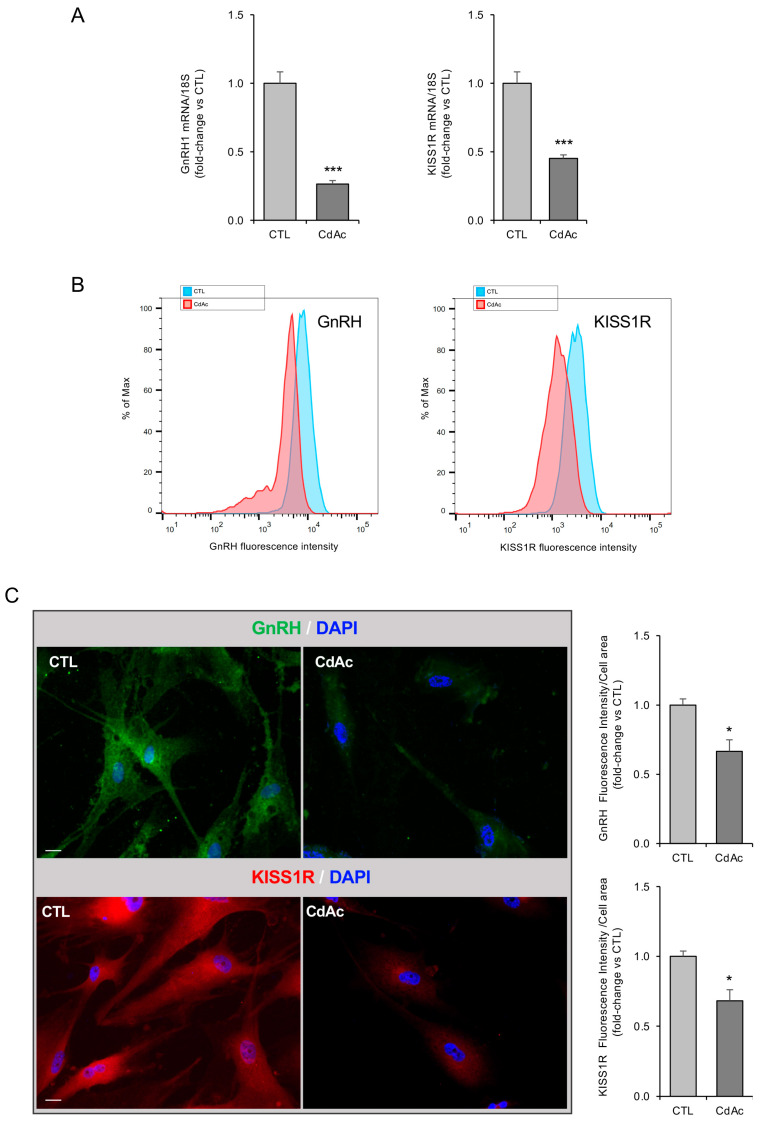
Effects of Cd on GnRH neuron phenotype. (**A**) mRNA expression of GnRH lineage markers *GNRH1* and *KISS1R* in untreated (CTL, light-gray box) and CdAc-treated (10 μM for 24 h; dark-gray box) FNCB4 cells. Data were normalized over the 18S ribosomal RNA subunit, used as the housekeeping gene. Results are expressed as fold change vs. untreated cells (CTL) and reported as means ± SEM (*n* = 9). *** *p* < 0.001 vs. CTL. (**B**) Representative overlaid histogram showing the fluorescence intensity for GnRH and KISS1R protein in control (untreated cells, CTL, light-blue peak) and CdAc-treated (10 μM for 24 h, red peak) cells, as detected in FNCB4 cells by flow cytometry analysis. (**C**) Representative immunofluorescence images and quantification analysis of GnRH (green) and KISS1R (red) in untreated (control, CTL) and CdAc-exposed FNCB4 (10 µM) cells for 24 h. DAPI-counterstained nuclei. Results are expressed as fold change vs. untreated cells (CTL) and reported as means ± SEM (*n* = 3). * *p* < 0.05 vs. CTL. Scale bars: 20 µm.

Notably, FNCB4 cells exposed to CdAc showed a significant reduction in both GnRH1 and KISS1R mRNA expression (both *p* < 0.001; Figure 8A). Immunofluorescence analysis confirmed a significant decrease in GnRH and KISS1R protein levels in CdAc-treated cells (both *p* < 0.05 vs. control; Figure 8C), also supported by FACS analysis (Figure 8B).

## 3. Discussion

In the present study, we demonstrated for the first time that Cd may directly affect mechanisms crucially involved in the migratory ability of human GnRH neuroblasts (FNCB4), thus suggesting that fetal exposure to Cd may compromise GnRH system development with deleterious consequences for puberty onset and fertility.

According to a recent report from the WHO, one in six people worldwide, around 17.5% of the reproductive-age population, is affected by infertility issues, evidencing the urgent need to increase research in this field [41]. While lifestyle factors are involved, increasing evidence points to environmental pollution as a crucial contributor to this trend [42]. The general population is chronically exposed to thousands of toxic chemicals, many of which act as EDCs capable of interfering with reproductive processes. Most research has focused on peripheral effects, particularly at the gonadal level, where EDCs have been shown to impair spermatogenesis and follicular development [43]. However, the possibility that environmental toxicants disrupt neuroendocrine circuit formation during critical developmental windows remains underexplored. Environmental exposure to Cd has been clearly recognized as a risk factor for reproductive dysfunction due to its gonadotoxicity. However, the effects of Cd on the neuroanatomical development of human hypothalamic GnRH neurons are not fully understood.

Taking advantage of the FNCB4 human cellular model of GnRH neuroblasts, we demonstrated that exposing cells to environmentally relevant concentrations of Cd impaired cytoskeletal remodeling, electrical activity, and intercellular communication, all essential processes for GnRH neuron development and function. For this in vitro study, we selected a dose of 10 µM CdAc as a subtoxic concentration that did not significantly affect FNCB4 cell viability, as confirmed by MTT assay. This concentration is consistent with previous investigations in human neuronal models [32,33,34], and reflects chronic exposure conditions representative of human bioaccumulation. Indeed, given the long biological half-life (up to 30 years) [44] of Cd and its ability to accumulate in body tissues for decades, toxicological in vitro studies indicated that concentrations relevant for mimicking Cd-mediated damage of tissues or body compartments ranged from 1 to 10 µM [45].

Consistent with the well-known ability of Cd to induce cellular oxidative stress and neurotoxicity [39], also in hypothalamic neurons [19], FNCB4 cells were able to activate oxidative stress mechanisms in response to Cd treatment, as demonstrated by a Cd-induced diffuse cytochrome C distribution, consistent with mitochondrial membrane permeabilization. This process is known to be often triggered by Cd-induced ROS in neuronal cells [46,47]. Moreover, Cd promoted inflammatory processes in FNCB4 cells by inducing a sixfold increase of COX2 mRNA expression and therefore creating a non-physiological inflammatory microenvironment that could be deleterious for GnRH neuron function. Indeed, we recently demonstrated how exposure to the proinflammatory cytokine TNFα impaired GnRH secretion in hypothalamic fetal cells [48]. From a mechanistic point of view, the most relevant finding of our study is the demonstration that FNCB4 cells responded to Cd exposure with significant alterations in cell morphology and phenotype, accompanied by functional changes collectively indicative of impaired neuronal maturation. In particular, Cd treatment affected the cytoskeletal architecture of the cells, inducing actin disassembly and fragmentation. As is well known, cytoskeletal structure and its remodeling are essential for cell migration, a key event occurring during GnRH neuron development. Fragmentation of F-actin might impair all the actin-dependent events, such as the actin polymerization-dependent leading process extension or the actin–myosin contraction at the rear end of the cell [49,50]. We also reported that Cd exposure induced a clear reduction in vinculin expression at the plasma membrane that may result in loss of focal adhesions implicated in the cyclic attachment and detachment of cells from the matrix. These events need to be synchronized with cytoskeletal reorganization to enable correct cell migration through “saltatory movement” [21,51]. The disruption of both focal adhesion dynamics and cytoskeletal organization likely represents the mechanism by which Cd impairs the migratory ability of FNCB4 cells, as demonstrated by the inhibition of cell migration detected with the wound-healing functional assay. Consistently with these findings, we also demonstrated that Cd affected the expression of genes related to the migration pathway, including ETB, FGFR1, NRP2, and NCAM1. It is well known that FGFR1 mutations cause genetic disorders associated with hypogonadotropic hypogonadism with GnRH deficiency and anosmia indicative of GnRH neuron maturation defects [52]. A lack of NRP2 has also been linked to alterations of the normal migratory process of GnRH neurons [53]. Alterations in FGFR1 are among the most frequently identified genetic defects in patients with congenital hypogonadotropic hypogonadism (CHH), both in normosmic forms and in Kallmann syndrome, and are directly linked to impaired GnRH neuron development and reproductive dysfunction, including delayed puberty and infertility [54]. Similarly, NRP2 mutations or dysregulation have been implicated in GnRH neuronal migration defects that underlie CHH phenotypes, further supporting the role of these genes in human reproductive pathophysiology. Accordingly, we demonstrated that Cd exposure significantly reduced ETB, FGFR1, and NRP2 mRNA expression. In contrast, Cd significantly increased the mRNA expression of NCAM1, a cell adhesion molecule involved in developmental processes and known to be expressed by GnRH neuron progenitors to guide the migratory pathway towards the brain [55,56,57]. We can speculate that the high expression of NCAM1 may hinder normal GnRH motility along the migratory route, favoring cell attachment to the matrix. In this regard, a study has demonstrated that upregulation of NCAM1 is associated with inhibition of invasiveness and migration of ameloblastoma cells [58]. In the context of reproductive health, polymorphisms and altered glycosylation of NCAM1 have been associated with impaired GnRH neuron migration and idiopathic cases of pubertal failure and infertility.

Concerning the mechanism through which Cd affects actin dynamics, we found that Cd-induced cytoskeletal changes appeared to be independent of the RhoA/ROCK pathway, since Cd did not affect RhoA membrane localization compared to untreated cells. This suggests that Cd may act downstream of RhoA activation, likely interfering more directly with actin cytoskeletal remodeling mechanisms. Consistent with our findings, it has been previously reported that Cd treatment affected actin regulatory protein in Sertoli cells, as manifested by truncation and depolymerization of actin microfilaments, thus determining changes in the localization of cell adhesion proteins, as well as alterations of the actin-based cytoskeleton [59].

The actin-based structural changes detected in FNCB4 cells after Cd treatment are also consistent with the alterations observed in terms of cell-to-cell communication. It is well known that cell-to-cell interactions between GnRH neurons are critical for their synchronized activity, which is essential not only during the migration route but also during their mature activity for allowing the typical pulsatile release of GnRH [60]. These interactions are essential for regulating the electrical activity of the cells through changing calcium homeostasis and activating intracellular signaling pathways involved in cell motility and functional maturation. Gap junctions (GJs), the most common type of cell-to-cell communication between GnRH neurons, enable small-molecule passage and current flow, which is the basis for synchronized depolarization inputs [61]. In this regard, we demonstrated a loss of GJ structure in Cd-treated FNCB4 cells, as revealed by a decrease in CX43 at the cell contacts. Accordingly, a significant reduction in gap-junctional current flow after Cd exposure was detected by electrophysiological measurements, further confirming defective cell-to-cell communication. These results, taken together with electrophysiological recordings of membrane passive properties, suggested that cells exposed to Cd undergo deep morphological changes that resulted in increased Cm, indicative of a wider cellular surface or cell swelling [62]. Disassembly of the actin cytoskeleton can even be the result of possible cell swelling caused by cell permeabilization to ions or osmotic alterations [63]. In line with this, changes in the actin cytoskeleton have been described during cell swelling and regulatory volume decrease in cultured astrocytes [64]. Indeed, reduction in Rm and RMP in Cd-treated FCNB4 cells is suggestive of an altered membrane permeability to ions with consequent changes in membrane potential. Accordingly, we recorded RMP depolarization, indicating an extra-cation influx at resting conditions after Cd exposure. Cd-induced reduction in K^+^ currents that have a role in action potential generation and membrane repolarization also supports this event. Based on these observations, we can hypothesize that the reduction in the overall K^+^ current could be due to a Cd-related impairment of Ca-activated K^+^ (KCa) channels because Cd treatment hampered/antagonized Ca^2+^ entry [65,66]. However, although Cd affects ion homeostasis and may interfere with the activity of Ca^2+^ and K^+^ channels, direct evidence linking Cd antagonism of Ca^2+^ entry to specific inhibition of KCa channels is lacking. Further research is thus needed to clarify this possible interaction.

From a mechanistic point of view, all the effects of Cd observed in this study can be explained based on its chemical nature and capacity to mimic divalent ions, such as calcium, which participate in physiological processes. Within the cell, Cd can interfere with calcium-dependent signaling cascades, thereby affecting transcription factors and gene expression and leading to altered intracellular pathways. Additionally, the impact of Cd on FNCB4 electrical activity could be explained by its ability to compete with other divalent cations with respect to transmembrane channels, either in terms of affinity or concentration, thereby interfering with intracellular signaling pathways. We can speculate that the electrophysiological changes induced by Cd may underlie the deleterious morphofunctional consequences observed in FNCB4 cells. Indeed, neuronal electrical activity is closely linked to functional outcomes, including cell migration, either through direct effects on cytoskeleton dynamics or indirectly by modulating gene expression profiles. In both processes, calcium represents the second main messenger, able to link neuron excitability to cellular response at the cytoskeleton and/or nucleus level. Consistent with this hypothesis, direct mechanisms in which calcium is involved in neuronal motility have been well characterized in many types of migrating neurons [67], including GnRH neurons [68].

In addition to impaired migratory ability, Cd exposure induced changes related to GnRH phenotype, as demonstrated by a significantly reduced expression of cell type-specific markers in FNCB4 cells, such as GnRH1 and KISS1R, also confirmed by FACS analysis and immunofluorescence. Considering the essential role of GnRH and kisspeptin signaling in the control of gonadotropin release and reproductive function, the downregulation of these markers strongly supports a mechanistic link between Cd exposure and impaired fertility potential.

To the best of our knowledge, this is the first time that these effects have been described in GnRH neurons of human origin. A similar inhibitory effect of Cd on GnRH mRNA expression was previously observed in prepubertal female rats [19]. In addition, a study in adult female rats reported that Cd exposure altered hypothalamic gene expression, causing a significant reduction in KISS1R mRNA [69]. Given the crucial physiological role played by the kisspeptin/KISS1R pathway in regulating GnRH neuron activity and thereby in maintaining a functional HPG axis [70], further investigations are needed to corroborate our findings.

## 4. Materials and Methods

### 4.1. Cell Cultures and Reagents

All experiments were carried out using human GnRH neuroblasts (FNCB4), a long-term cell culture established and cryogenically preserved as previously described [28]. The use of fetal material for research purposes was approved by the National and Local Ethics Committee of the University of Florence (protocol 678304).

FNCB4 cells were cultured at 37 °C in a humified 5% CO_2_ atmosphere in Coon’s modified Ham’s F12 medium (Sigma-Aldrich, St. Louis, MO, USA) supplemented with 10% fetal bovine serum (FBS; Euroclone, Milan, Italy). Each experiment was performed in serum-free Coon’s modified Ham F12 medium after 24 h of starvation.

Cadmium acetate (CdAc) was supplied by Sigma-Aldrich and dissolved in distilled sterile water at 100 mM concentration as stock solution. FNCB4 cells were treated with 10 µM of CdAc for 24 h and then processed as appropriate. Cell viability was evaluated by treating the cells with 5, 10, 30, 50, and 100 µM of CdAc for 24 h. All experiments were performed with at least three independent biological replicates.

### 4.2. Immunocytochemistry

The immunofluorescence analysis on FNCB4 cells was performed as previously described [71]. Briefly, cells were fixed in paraformaldehyde 3.7% in phosphate-buffered saline (PBS, Sigma-Aldrich), permeabilized with Triton X-100 0.1% (Sigma-Aldrich) in PBS, and incubated with bovine serum albumin 1% (BSA, Sigma Aldrich). Immunofluorescence staining was carried out by incubating overnight at 4 °C the following primary antibodies: rabbit polyclonal anti-GnRH (1:100, Proteintech, Manchester, UK), rabbit polyclonal anti-KISS1R (1:100; Phoenix Pharmaceuticals Inc., Burlingame, CA, USA), rabbit polyclonal anti-cytochrome C (1:200, Santa Cruz Biotechnologies, Dallas TX, USA), mouse polyclonal anti-vinculin (1:200, Sigma Aldrich), rabbit polyclonal anti-connexin 43 (1:200, Sigma Aldrich), and mouse polyclonal anti-RhoA (1:100, Santa Cruz Biotechnologies). The day after, samples were incubated with Alexa Fluor 488-conjugated or Alexa Fluor 568-conjugated anti-mouse or anti-rabbit secondary antibodies (1:200, Molecular Probes, Eugene, OR, USA), as appropriate. Alexa Fluor 488-conjugated phalloidin (1:200, Molecular Probes) was incubated for 1 h at room temperature and used for cytoskeletal F-actin detection. To visualize membrane colocalization, cells were stained with WGA (wheat-germ-agglutinin, rhodamine-conjugated, 1:250; Vector Laboratories, Newark, CA, USA) for 10 min before fixing. ProLong Gold antifade reagent with 4′-6′-diamidino-2-phenylindole dihydrochloride (DAPI; Molecular Probes) was used for mounting slides and counterstaining nuclei. For negative controls, we avoided primary antibody incubation.

Quantitative RhoA/WGA co-localization was evaluated using the JACoP plugin in Fiji (https://imagej.net/Fiji, accessed on 12 October 2023), with Pearson correlation coefficient (PCC) values computed above threshold across the Z-stack. PCC values range from +1 (perfect correlation) to −1 (inverse correlation), with 0 indicating no correlation.

Quantitative analysis of GnRH and KISS1R immunofluorescence images was performed using Fiji (https://imagej.net/Fiji). Mean fluorescence intensity was measured for each image and normalized to the area occupied by cells, calculated by applying a consistent threshold.

Images were analyzed and collected using a Stellaris 5 confocal microscope (Leica, Wetzlar, Germany) from the Department of Experimental and Clinical Medicine Imaging Platform of the University of Florence.

### 4.3. Flow Cytometry

Cells were analyzed by flow cytometry as already reported [72]. Briefly, 2 × 10^5^ FNCB4 cells were resuspended in PBS supplemented with 1% FBS, and after fixation with paraformaldehyde 2% in PBS, incubated with anti-GnRH (1:100; Proteintech) and anti-KISS1R (1:150; Alomone Labs, Jerusalem, Israel) primary antibodies. Alexa Fluor 488 goat anti-rabbit IgG (1:200; Molecular Probes) was used as a secondary antibody. For negative controls, we avoided primary antibodies. Stained cells were analyzed using a FACS CantoII flow cytometer (Becton-Dickinson, San Jose, CA, USA). Data were analyzed using BD FACSDiva Software v9.0 (BD) (Becton-Dickinson, Franklin Lakes, NJ, USA) and FlowJo v10 (Tree Star Inc., Ashland, OR, USA).

### 4.4. RNA Extraction and Quantitative RT-PCR Analysis

Isolation of total RNA from 2 × 10^5^ FNCB4 cells was carried out as previously reported [48] using an RNeasy mini kit (Qiagen, Hilden, Germany) according to the manufacturers’ instructions. cDNA synthesis was performed using an iScript^TM^ cDNA synthesis kit (Bio-Rad Laboratories, Hercules, CA, USA). Genes were analyzed by quantitative real-time RT-PCR (qRT-PCR) using SsoAdvanced Universal SYBR^®^ Supermix and the CFX Duet Real-Time PCR Detection System (both Bio-Rad Laboratories). Specific probes and primers for target genes were predeveloped assays (Life Technologies, Carlsbad, CA, USA) or custom-made by sequences available at NCBI GenBank (https://www.ncbi.nlm.nih.gov/). The 18S ribosomal RNA subunit was used as the housekeeping gene, quantified with a predeveloped assay (Hs99999901_s1, Life Technologies) and taken as relative quantitation of the target genes based on the comparative threshold cycle (Ct) 2^−ΔΔCt^ method [73], as it shows stable expression even under stress conditions, including heavy metal exposure [74].

### 4.5. MTT Assay

Cadmium cytotoxicity was evaluated by MTT assay (Sigma-Aldrich) as previously reported [75]. Briefly, 5 × 10^3^ FNCB4 cells per well were seeded in 96 multi-well plates. After 24 h, cells were serum-starved for 8 h and then treated with different concentrations of CdAc (0, 5, 10, 30, 50, and 100 µM) for an additional 24 h. Next, culture medium was replaced and 10 μL of MTT solution added for 3 h at 37 °C. Optical density was measured by a Multiscan FC spectrophotometer (Thermo Fisher Scientific, Waltham, MA, USA) with the filter set to 450 nm. Cell viability is reported as a percentage of control (untreated cells), taken as 100%. The range (0–100 µM) of CdAc concentrations tested was selected based on studies in neuronal cells, where cadmium sensitivity varies by cell type and assay [35,37,76,77]. This approach was useful to define the cytotoxicity profile of the FNCB4 model and identify 10 µM as a subcytotoxic dose.

### 4.6. Electrophysiological Records

FCNB4 cells’ biophysical properties were analyzed by the whole-cell patch-clamp technique, as previously described [71]. The cells were constantly superfused with a physiological external solution with the following composition (mM): 150 NaCl, 5 KCl, 2.5 CaCl_2_, 1 MgCl_2_, 10 D-glucose, and 10 HEPES (pH = 7.4). To fill the patch pipettes, we used the following internal solution (mM): KCl 130, NaH_2_PO_4_ 10, CaCl_2_ 0.2, EGTA 1, MgATP 5, and HEPES/KOH 10 (pH = 7.2). The pipette resistance was around 1–2 MΩ. The setup for electrophysiological measurements was as previously reported [78] and consisted of the Axopatch 200 B amplifier (Axon Instruments, Union City, CA, USA), an analog-to-digital/digital-to-analog interface (Digidata 1200; Axon Instruments), and pClamp software version 6.0 (Axon Instruments). Currents were low-pass filtered at 1 kHz with a Bessel filter. By applying a stimulus of I = 0 nA in current-clamp mode, we recorded the resting membrane potential (RMP). To analyze the membrane passive properties, namely the cell linear capacitance (Cm) and cell resistance (Rm), we applied a voltage pulse protocol in voltage-clamp mode starting from a holding potential (HP) of −70 mV by applying two 75 ms step voltage pulses to −80 and −60 mV, as detailed in a previous paper [71]. The Cm value is related to the cell surface area, considering the specific Cm = 1 μF/cm^2^. Rm is indicative of the membrane permeability. To estimate the voltage-dependent ion current appearance, the cells were held at HP = −60 mV and voltage steps for 1 s ranging from −80 to 50 mV were applied in 10 mV increments. Passive currents were removed directly online using the P/4 procedure. The current amplitude size was regularly normalized to Cm to enable a proper comparison of the currents recorded from cells of different dimensions and is reported as current density (I/Cm). For the gap junctional current evaluation, we used the voltage-clamp protocol of stimulation, as previously reported [79]. In brief, cell 1 of the pair was stepped from a holding potential (HP) of 0 mV using a bipolar 5 s pulse protocol starting at transjunctional voltage Vj = ±10 mV and ongoing at 20 mV increments up to ± 150 mV. The transjunctional current flowing through GJs is indicated as I_j_. Specifically, the amplitude of I_j_ determined at the peak was named I_j,inst_ (instantaneous transjunctional current), whereas that measured at the end of each pulse is indicated as I_j,ss_ (steady-state transjunctional current).

### 4.7. Statistical Analysis

Data are expressed as means ± standard error of the mean (SEM) or standard deviation (SD), as appropriate. To estimate the normal distribution of data, we used the Shapiro–Wilk test. Student’s unpaired *t*-test was performed to determine statistical significance, which was defined as *p* < 0.05. In cases where data did not follow a normal distribution, the nonparametric Mann–Whitney U test was applied. In the electrophysiological experiments, n represents the number of cells analyzed. Data were analyzed using the Statistical Package for the Social Sciences (SPSS v.28.0; SPSS Inc., Chicago, IL, USA; https://www.ibm.com/support/pages/downloading-ibm-spss-statistics-28).

## 5. Conclusions

In conclusion, our results demonstrate for the first time that Cd can interfere with the migratory ability of human GnRH neuroblasts by affecting intracellular pathways involved in cytoskeletal remodeling and focal adhesion dynamics. Moreover, Cd impaired FNCB4 electrical properties and crucial functions related to cell-to-cell communication, thereby potentially contributing to neurodevelopmental defects. Although derived from an in vitro study, which can suffer from limitations, we believe that—being obtained in an authentic, non-immortalized human cellular model—these findings are relevant considering the obvious difficulty in replicating the experiments in vivo in the human organism. Similar in vivo studies in animal models would not have the same relevance.

Overall, our findings provide new insights into the comprehension of mechanisms through which Cd, acting at the central level on GnRH neuron development, may interfere with human reproductive function. Our study highlights the urgent need for strengthening public health strategies aimed at minimizing exposure to environmental neurotoxicants, especially during critical windows of fetal brain development.

## Data Availability

Data will be made available on request.

## Supplementary Materials

The following supporting information can be downloaded at: https://www.mdpi.com/article/10.3390/ijms27031221/s1.
