# Supplementary figures and images for: Cadmium Impairs Human GnRH Neuron Development: Mechanistic Insights into Reproductive Dysfunction
Source: Int J Mol Sci. 2026 Jan 26;27(3):1221. doi: 10.3390/ijms27031221 (PMC12897737; doi:10.3390/ijms27031221)

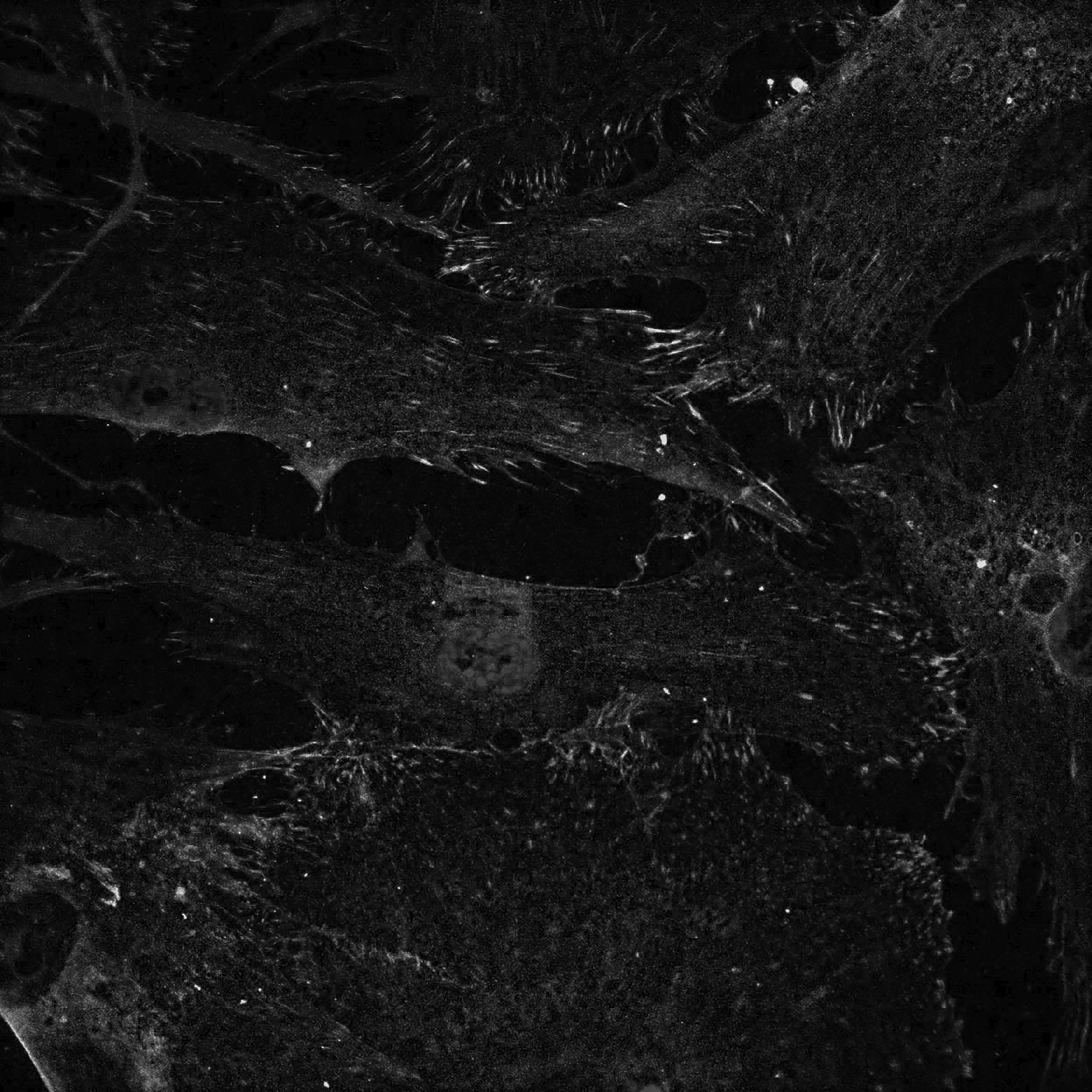

Supplement: Supplementary file 1 [file ijms-27-01221-s001.zip › Supplementary_material_1/Fig4B_Ctl_raw_Grayscale.tif]

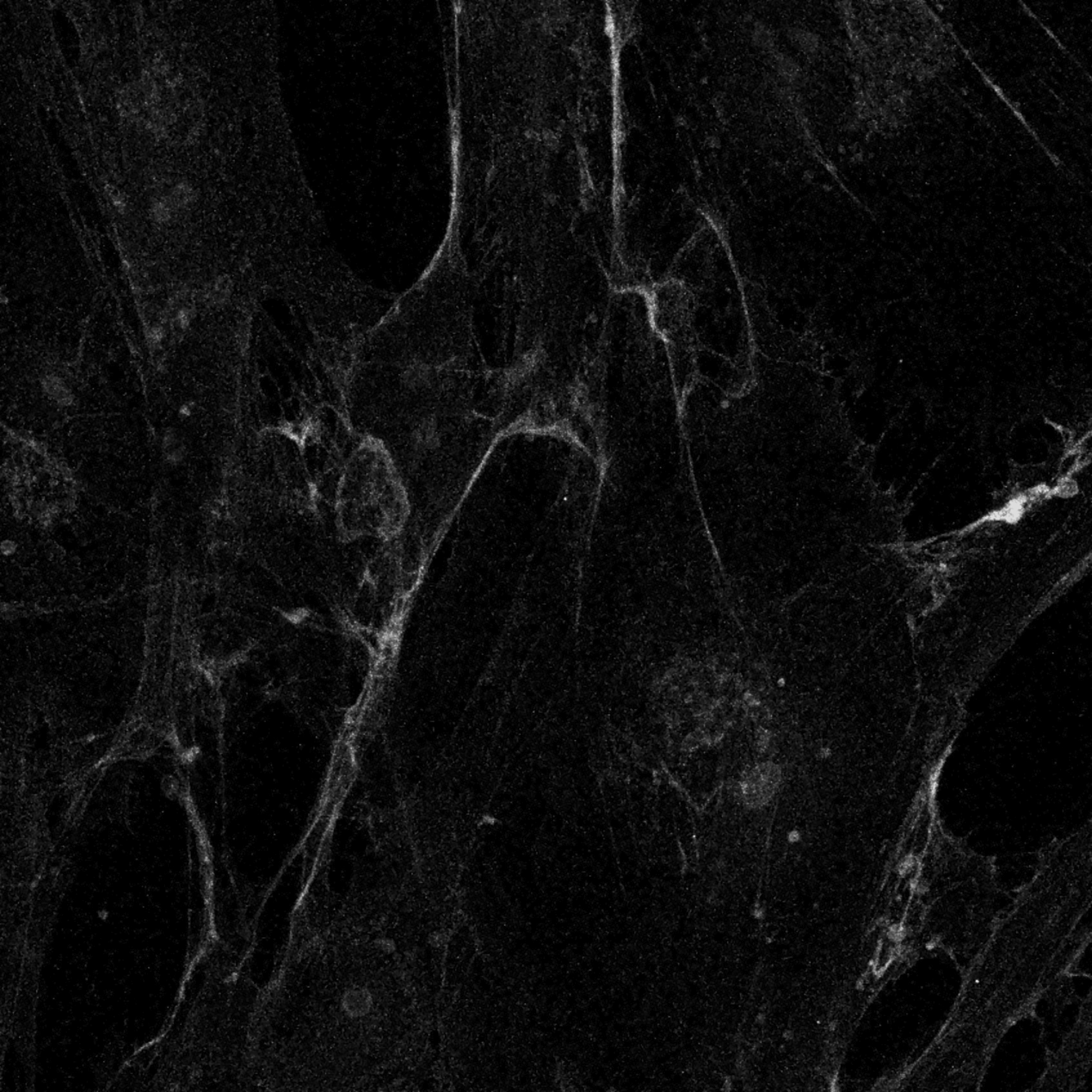

Supplement: Supplementary file 1 [file ijms-27-01221-s001.zip › Supplementary_material_1/Fig4C_Cd_RhoA_raw_Grayscale.tif]

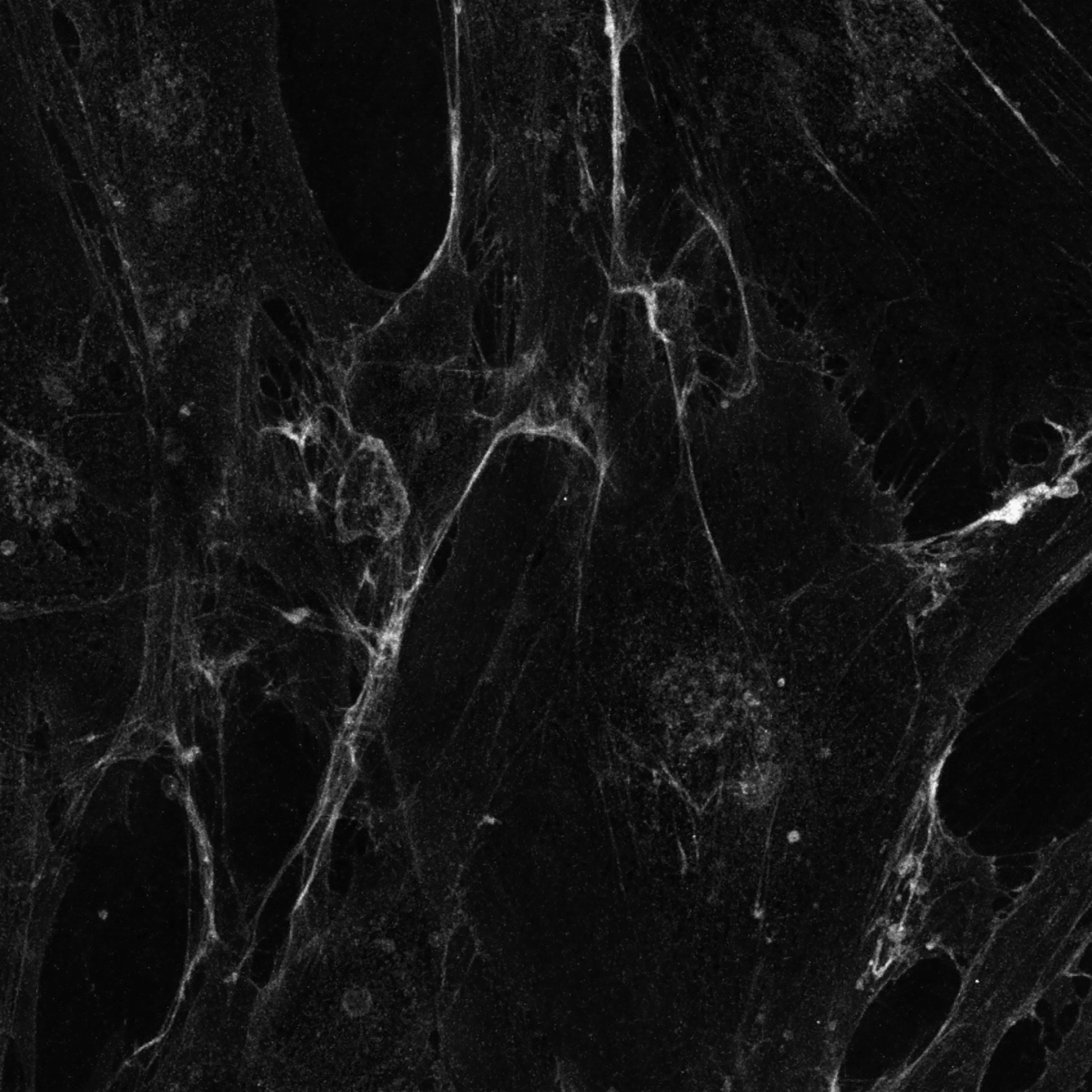

Supplement: Supplementary file 1 [file ijms-27-01221-s001.zip › Supplementary_material_1/Fig4C_Cd_Composite_raw_Grayscale.tif]

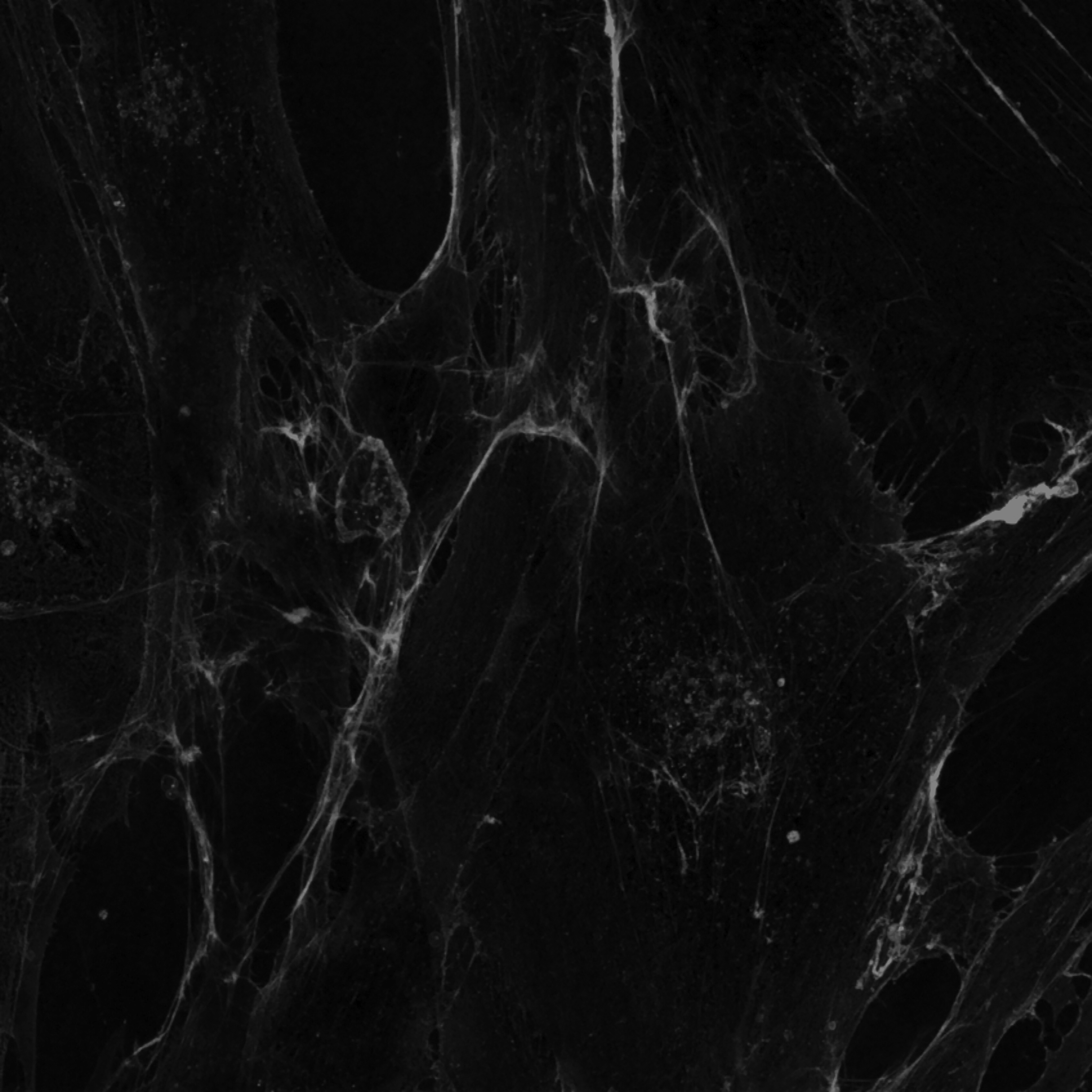

Supplement: Supplementary file 1 [file ijms-27-01221-s001.zip › Supplementary_material_1/Fig4C_Cd_wga_raw_Grayscale.tif]

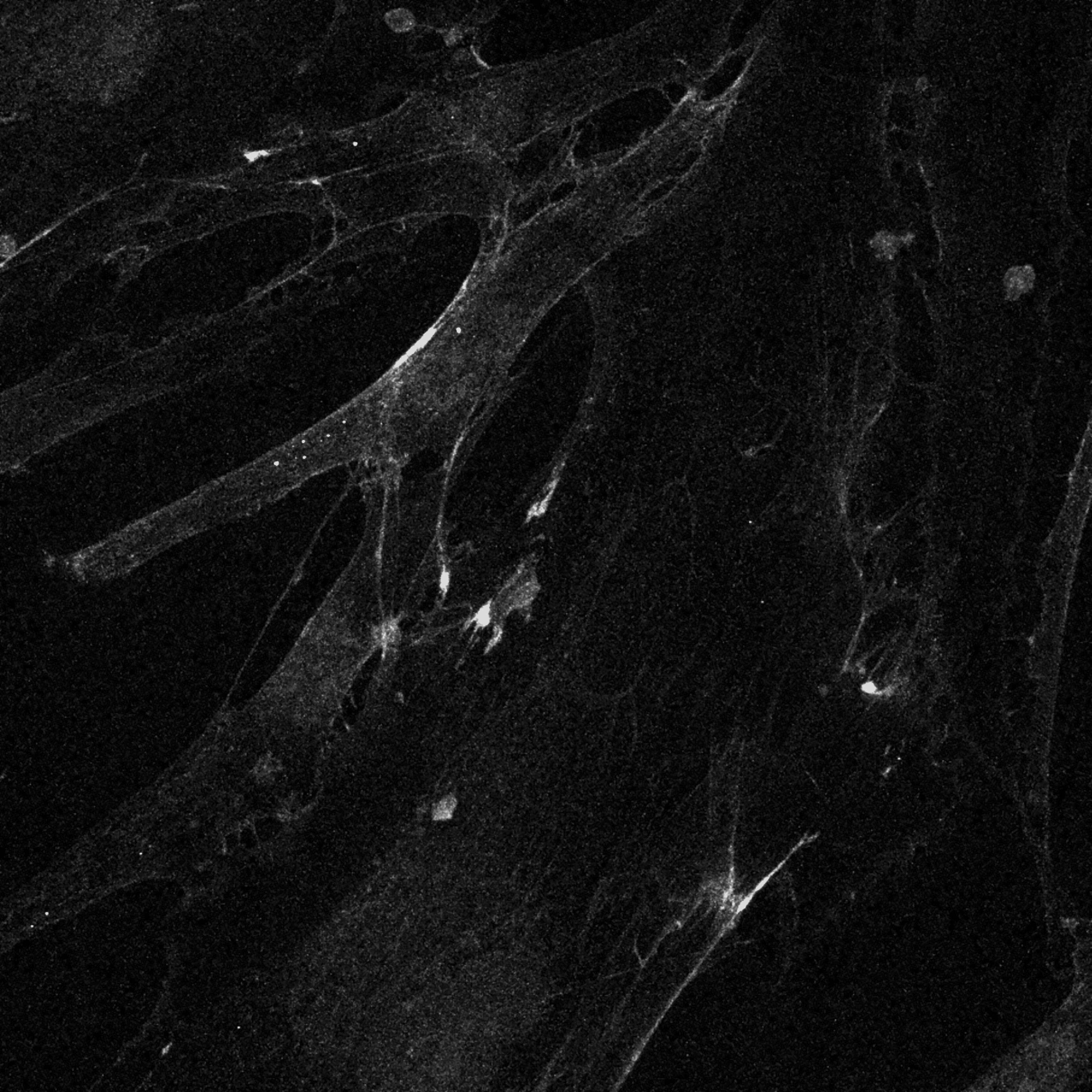

Supplement: Supplementary file 1 [file ijms-27-01221-s001.zip › Supplementary_material_1/Fig4C_Ctl_RhoA_raw_Grayscale.tif]

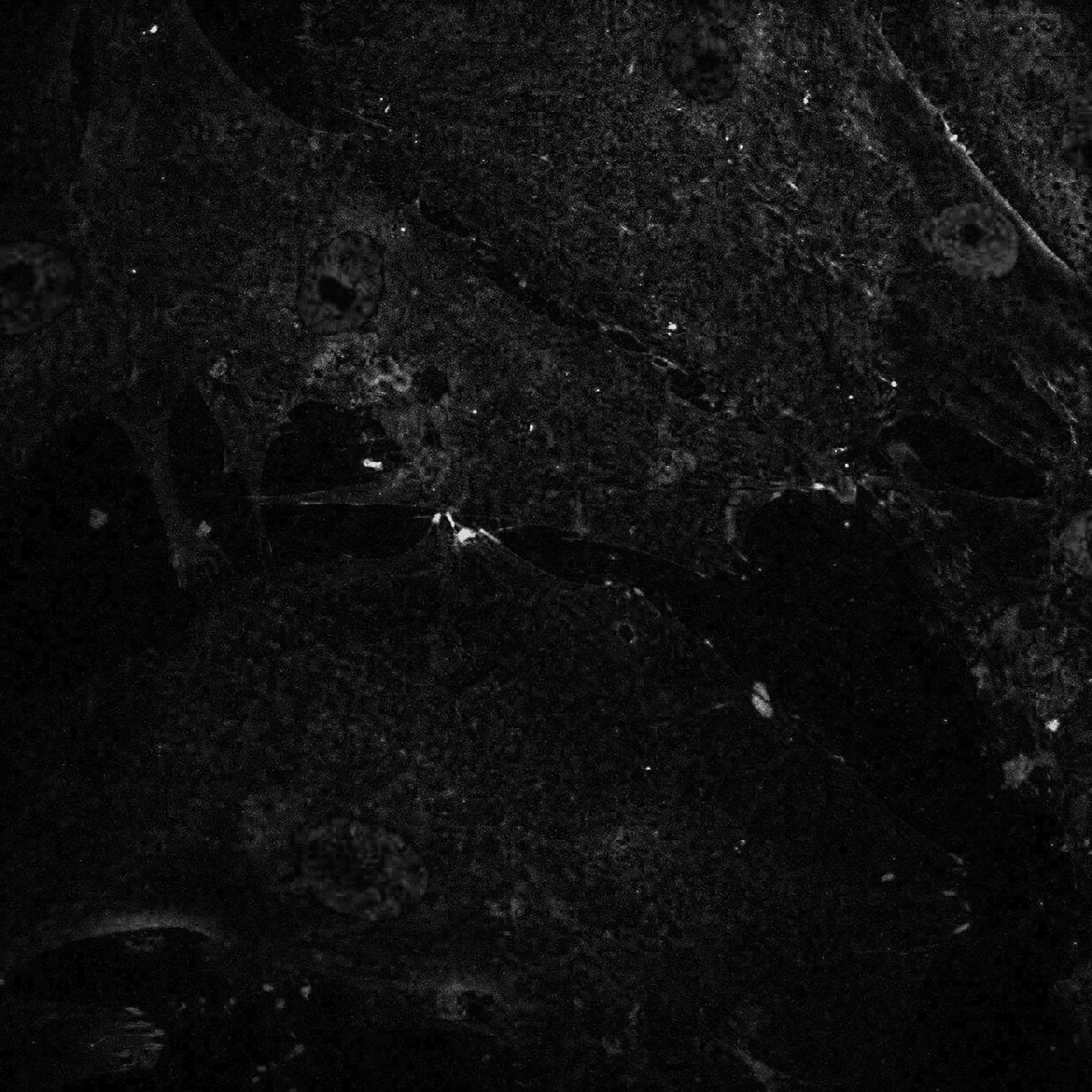

Supplement: Supplementary file 1 [file ijms-27-01221-s001.zip › Supplementary_material_1/Fig4B_Cd_raw_Grayscale.tif]

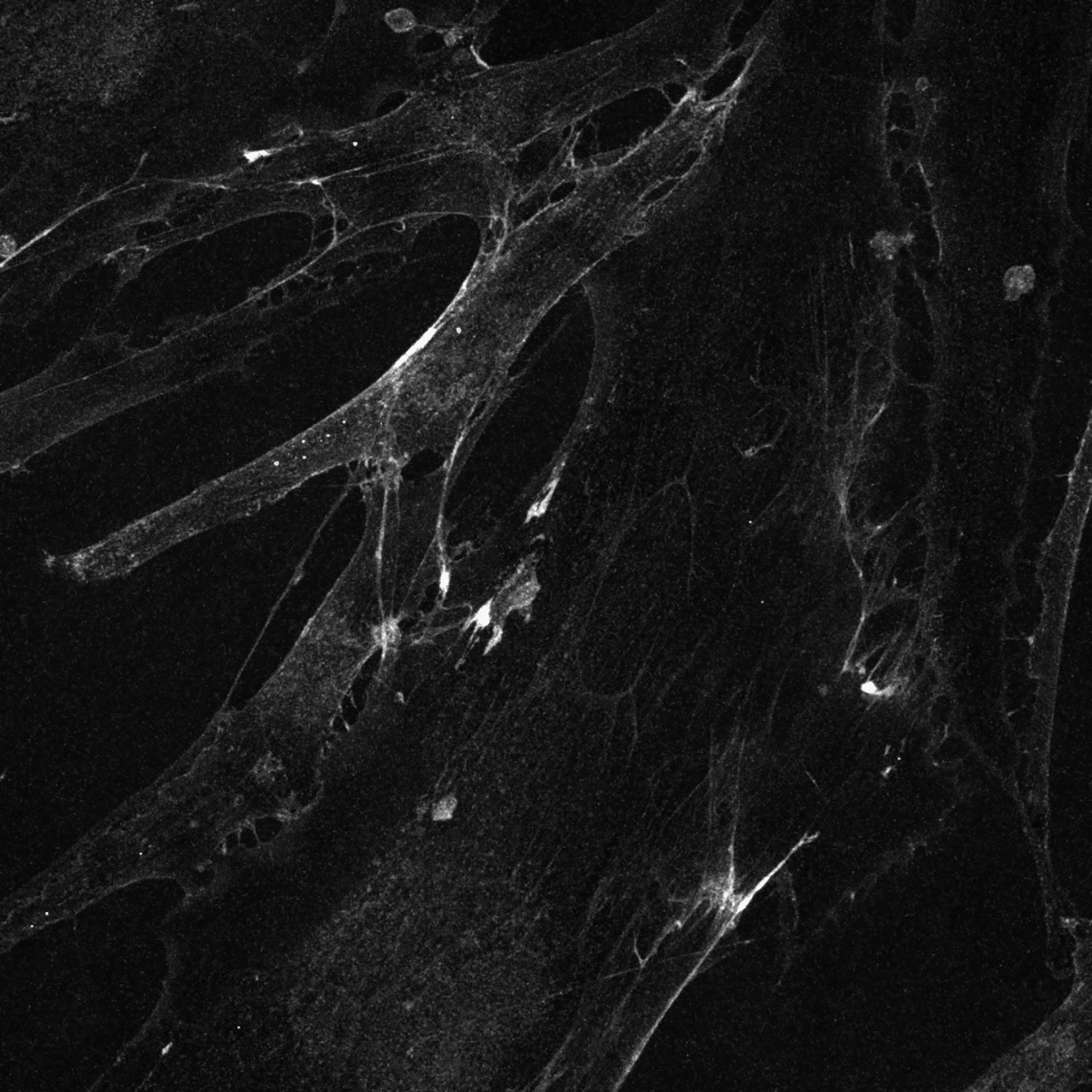

Supplement: Supplementary file 1 [file ijms-27-01221-s001.zip › Supplementary_material_1/Fig4C_Ctl_Composite_raw_Grayscale.tif]

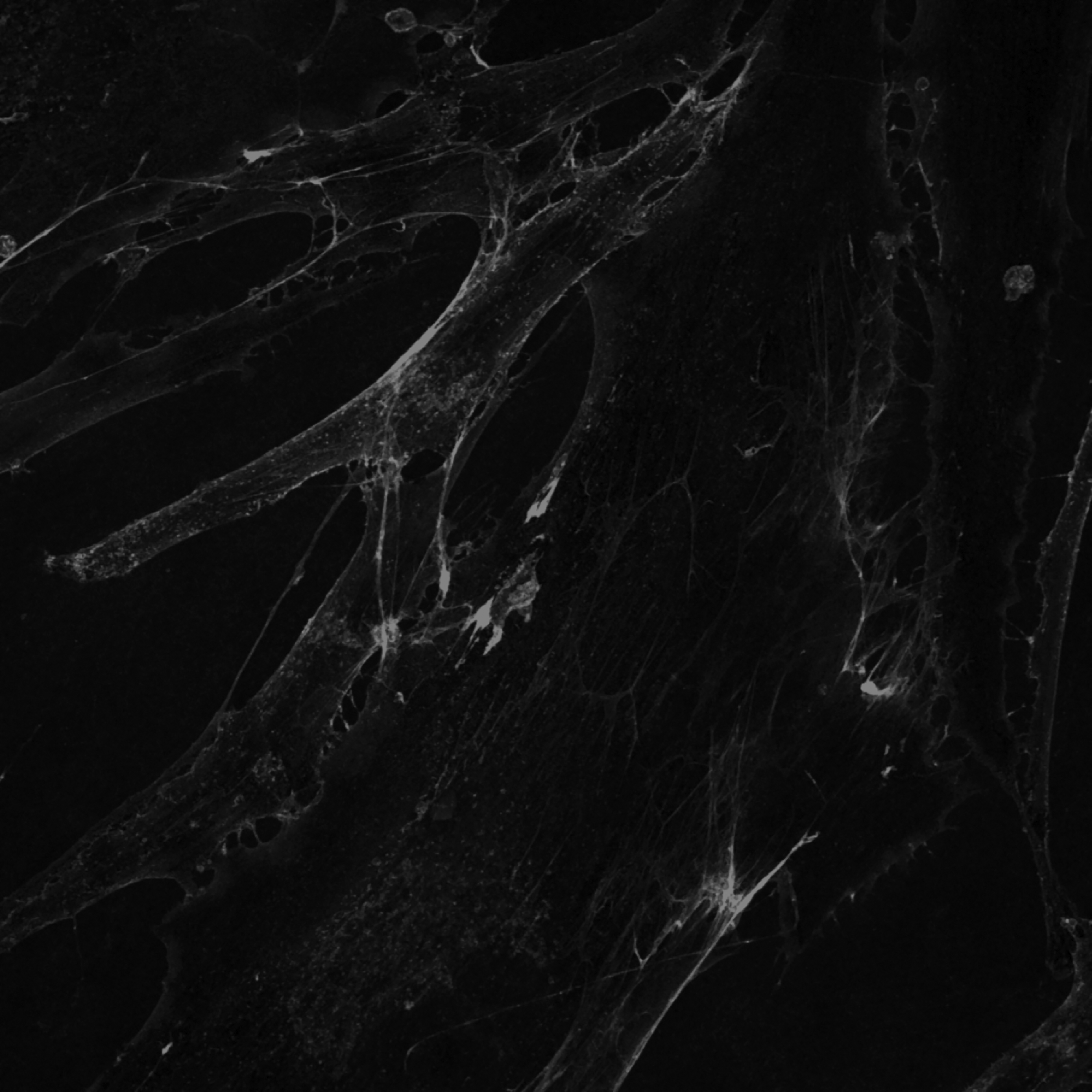

Supplement: Supplementary file 1 [file ijms-27-01221-s001.zip › Supplementary_material_1/Fig4C_Ctl_wga_raw_Grayscale.tif]
